# Facial Nerve Function After Microsurgical Resection in Vestibular Schwannoma Under Neurophysiological Monitoring

**DOI:** 10.3389/fneur.2022.850326

**Published:** 2022-05-24

**Authors:** Felix Arlt, Johannes Kasper, Dirk Winkler, Katja Jähne, Michael Karl Fehrenbach, Jürgen Meixensberger, Caroline Sander

**Affiliations:** Department of Neurosurgery, University Hospital Leipzig, Leipzig, Germany

**Keywords:** vestibular schwannoma, intraoperative neurophysiological monitoring, facial nerve function, microsurgery in vestibular schwannoma, facial nerve EMG

## Abstract

**Background:**

The use of intraoperative neurophysiological monitoring, including direct nerve stimulation (especially the facial nerve), acoustic evoked potentials (AEP) and somatosensory evoked potentials (SSEP), is a helpful tool in the microsurgery of vestibular schwannoma to prevent nerve injury. Patient characteristics and intraoperative and postoperative variables might also influence the postoperative facial nerve function. The study was performed to investigate these variables and the intraoperative neurophysiological monitoring values.

**Methods:**

Seventy-nine patients with vestibular schwannoma were included consecutively into this study. Intraoperative neurophysiological monitoring, including SSEP, AEP, and direct nerve stimulation for facial and trigeminal nerve electromyography, was performed utilizing digital data storage in all cases. The intensity (in volts) of the direct stimulation and the latency (in ms) for the orbicularis oculi and the orbicularis oris muscle and the amplitude (in mV) was measured. Univariate and multivariate statistical analyses concerning the different parameters was performed directly after the operation and in the subsequent follow-ups 3 and 6 months after the operation.

**Results:**

The mean intensity was 0.79 V (SD.29). The latency and amplitude for the oris muscle was 5.2 ms (SD 2.07) and 0.68 mV (SD.57), respectively. The mean latency for the occuli muscle was 5.58 ms (SD 2.2) and the amplitude was 0.58 mV (SD 1.04). The univariate and multivariate statistical analyses showed significance concerning the postoperative facial nerve function and the amplitude of the direct stimulation of the facial nerve in the orbicularis oris muscle (*p* = 0.03), so repeated direct nerve stimulation might show FN function deterioration. The mean diameter of the tumors was 24 mm (range 10–57 mm). Cross total resection and near total was achieved in 76 patients (96%) and subtotal in three patients (4%). The preoperative House–Brakeman score (HBS) 1 was constant in 65 (82%) cases. The mortality in our series was 0%; the overall morbidity was 10%. The HBS was not influenced concerning the extent of resection. The mean follow-up was 28 months (range 6 to 60 months). The limitations of the study might be a low number of patients and the retrospective character of the study.

**Conclusion:**

Intraoperative neurophysiological monitoring is crucial in vestibular schwannoma surgery. Repeated direct nerve stimulation and a detected decreased amplitude might show facial nerve function deterioration.

## Introduction

Vestibular schwannoma (VS), historically also known as acoustic neuroma, is the most common, benign and sporadic tumor in the cerebellar pontine angle. The origin of the tumor are the Schwann cells of the vestibulocochlear nerve and the tumor growth is slow (1, 2). The tumor in neurofibromatosis type 2 patients occurs mostly bilaterally and is combined with other tumors in the central and peripheral nerve system, such as meningioma and astrocytoma. The treatment of sporadic VS consists of three strategies. The “wait and see/scan” strategy is preferred in small tumors with no mass effect and few symptoms (3, 4). Radiotherapy (stereotactic radiotherapy, Gamma knife, or Cyber knife radiosurgery) could be indicated for medium size tumors up to 2–3 cm in diameter, even in older patients with high anesthesiological risk factors (4, 5). The third treatment option is microsurgical removal of the tumor (6, 7).

The use of intraoperative neurophysiological monitoring, including direct facial and trigeminal nerve stimulation, AEP (acoustic evoked potentials) and SSEP (somatosensory evoked potentials), is a helpful tool in the microsurgery of VSs to prevent nerve injury (8). The electrical stapedius reflex testing for cochlear nerve monitoring during translabyrinthine resection of vestibular schwannoma is another option for intraoperative monitoring (9). The main goal of the operation is to resect as much tumor as possible without nerve injury to either hearing or facial nerve function. Both are accompanied by a decreased quality of life in the case of deterioration (10–14).

The aim of the actual study is to analyze factors which might influence facial nerve function after microsurgical resection of unilateral VS by using intraoperative neurophysiological monitoring.

## Patients and Methods

**Figure 1:**
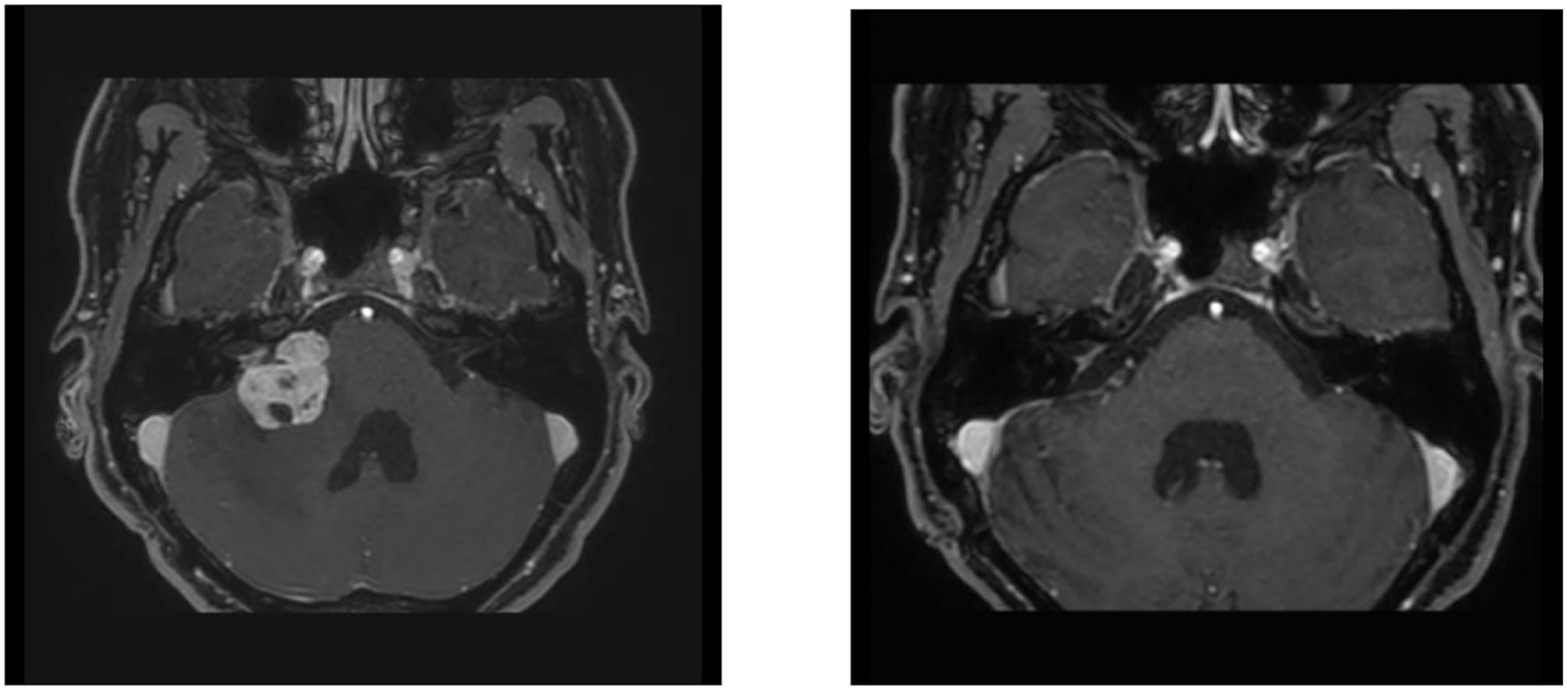
A 43-year-old female patient with a cystic vestibular schwannoma on the right. On the left the preoperative findings, on the right the findings 3 months after the resection. Microsurgical resection under intraoperative monitoring. At the beginning of the resection, the amplitude of the EMG was 1.3 mV and decreased to 0.48 mV toward the end of the operation.

Microsurgical resection due to VS was performed in 85 patients *via* a retrosigmoidal approach between 2015 and 2020. Six patients were lost in follow-up; therefore, 79 patients were included into the study. Patients suffering neurofibromatosis type II were excluded. The local ethic committee of the University Leipzig approved the study (reference: 145/21-ek). The patient data were analyzed retrospectively by using the electronic patient chart (SAP, Walldorf, Germany). Patient characteristics are shown in Table 1. The indication for the operation results from the patient's symptoms, for example increased hearing loss, dizziness, unsteady gait; also, for large tumors with a mass effect, compression of the fourth ventricle or tumor growth in the follow-up. In the event of recurrence growth in the follow-up or tumor growth after radiation, we make the indication for another operation. In our department the semi-sitting position is preferred. In this case, intraoperative continuous transesophageal echocardiography to detect air embolism is performed. In the case of a patent foramen ovale, the operation is implemented in the park bench position. The surgical philosophy is the microsurgical pial preparation of the tumor capsule with intermittent tumor reduction with the CUSA. In all cases, we use intraoperative neurophysiological monitoring, including SSEP, AEP, and direct nerve stimulation for facial and trigeminal nerve electromyography *via* the Inomed ISIS IOM System^®^, Emmendingen, Germany. Particularly the intensity (in volts) of the direct stimulation and the latency (in ms) for the orbicularis oculi and the orbicularis oris muscle as well as the amplitude (in mV) were measured. The digital storage of data was performed for each patient in a prospective manner. The House–Brackmann scale (HBS) documented facial nerve function immediately before the operation, directly after the operation, and subsequently after 3 and 6 months. Magnetic resonance imaging for resection control was performed 3 months postoperatively and subsequently once a year (Figure 1). Resection was described as cross total, near total (resection more than 95% of the tumor mass) and subtotal (resection ~95% of the tumor). We also documented intraoperative complications, such as air embolism in the semi-sitting position, and postoperative complications, such as hydrocephalus or rebleeding. The statistical data were analyzed using SSPS^®^ by IBM^®^.

**Table 1:** Patients' characteristics.

**Sex**	**Female 46**	**Male 33**
Age (years)	56	Range: 17–87
Location	Left 41	Right 38
Tumor size (mean diameter in mm)	24	Range: 10–57
Cystic tumor		16 (20 %)
Hannover classification	•T2 •T3 •T4a •T4b	3 (4 %) 29 (37 %) 19 (24 %) 28 (35 %)
Recurrent tumor		2 (3 %) 5 (6 %)
Preoperative radiotherapy		
Position	Semi-sitting Park bench	65 (82 %) 14 (18 %)
Extent of resection	•Total •Near total •Subtotal	31 (39 %) 45 (57 %) 3 (4 %)
Follow-up (MEAN in months)	28 Range: 6–60	
Pre-operative Hearing deteriortation		46 (58 %)
Hearing preservation		71 (90 %)
Preoperative HBS/Post-operative HBS/Follow up HBS	•HBS 1 75 (95 %)/65 (82 %)/65 (82 %) •HBS 2 1 (1 %)/ 7 (9 %)/ 5 (6 %) •HBS 3 3 (4 %)/ 4 (5 %)/ 4 (5 %) •HBS 4 0/ 3 (4 %)/ 5 (6%)	

## Results

The intraoperative monitoring values considering direct facial nerve stimulation are shown in Table 2. The facial nerve function was impaired in three patients before the operation, HBS 2 in one patient and HBS 3 in two patients. In these patients, the HBS was unchanged after the operation and follow-up. The preoperative HBS 1 was constant in 65 (82%) cases. We detected a deterioration in the facial nerve function postoperatively in 14 (18%) patients (HBS 2: seven patients, HBS 3: four patients, HBS 4: three patients). Even in these cases, loss of the pial border, stronger vascularization or tough tumor structure was described by the surgeon. A worsening in the facial nerve function in these patients was seen in the follow-up in two cases. We found no statistically significant correlation between the preoperative tumor size and the preoperative facial nerve function (*p* = 0.423). The tumor size and postoperative facial nerve function also showed no significant correlation (*p* = 0.343). The SSEPs showed no changes during the operation in any cases. The trigeminal nerve and the caudal cranial nerves were not affected in any case. A deterioration in hearing was observed in 46 patients (58%). In these cases, the hearing ability was already reduced preoperatively. A preservation of serviceable hearing was achieved in 71 patients (90%). In addition to the facial function, the preservation of the hearing function is the main goal of the resection. The preservation of the cochlearis fibers with the leaving behind of the tumor capsule is the op strategy.

**Table 2:** Values in direct facial nerve stimulation.

**Values in direct nerve stimualtion**	**Mean**	**Median**	**Standard deviation**
Intensity in voltS	0.76	0.7	0.29
Latency oris muscle	5.2	5.5	2.07
Amplitude oris muscle	0.68	0.58	0.57
Latency occuli muscle	5.58	5.2	2.2
Amplitude oculi muscle	0.85	0.67	1.04

*The intensity is given in volts (V), the latency is given in milliseconds (ms), and the amplitude is given in millivolts (mv)*.

The univariate statistical analyses showed significance concerning the postoperative facial nerve function and the amplitude of the direct stimulation of the facial nerve in the orbicularis oris muscle (*p* = 0.03). The direct postoperative HBS was also significantly influenced by the presence of a cerebrospinal fluid (CSF) leakage (postoperative HBS, *p* = 0.032); this could not be confirmed in the follow-up HBS (*p* = 0.216) (Table 3). These findings could also be confirmed in the binary multivariate regression analysis, while the other items, such as in the univariate analysis, showed no statistical significance. The HBS was not influenced concerning the extent of resection.

**Table 3:** Univariate and multivariate analyses for HBS post-op and HBS in the follow-up concerning preoperative, perioperative, and postoperative variables.

**Univariate analysis**	**HBS post-OP (*p*-value)**	**HBS follow-up (*p*-value)**
Extent of resection • Cross total • Near total • Subtotal	0.363 0.588 0.571	0.547 0.363 0.571
Age	0.186	0.186
Diameter (mm)	0.175	0.175
Recurrent tumor	0.999	0.999
Preoperative radiotherapy	0.890	0.890
Preoperative HBS	0.999	0.999
Operation position	0.711	0.711
Mib1_K67	0.838	0.838
Cystic tumor	0.397	0.397
Re-bleeding	0.134	0.066
CSF leakage	**0.032**	0.216
Post-operative HC	0.999	0.999
Intraoperative air embolism	0.483	0.483
Latency oris muscle	0.758	0.523
Latency oculi muscle	0.617	0.617
Amplitude oris muscle	**0.030**	**0.030**
Amplitude oculi Muscle	0.051	0.051
**Multivariate Analysis**
Amplitude oris muscle	**0.041**	**0.032**
CSF leakage	**0.045**	0.112

*Significant values are in bold*.

The mean follow-up was 28 months (range 6–60 months). The operation was performed due to a recurrent tumor, 14 months respectively 28 months after the first operation, in two of the 79 patients. Five patients were operated after a stereotactic radiotherapy because of ongoing tumor growth. A cystic tumor growth was described in 16 cases. The mean diameter of the tumors was 24 mm (range 10–57 mm). Sixty-five patients were operated in the semi-sitting position and 14 in the park bench position. Cross total resection was achieved in 31 patients (39%), near total (resection more than 90% of the tumor) in 45 patients (57%) and subtotal (resection <90%) in three patients (4%). The histopathological diagnosis was Neuroma WHO I° in all cases, and the mean Mib1 K67 index was 3% (range 1–6%). After incomplete resection, six (11%) patients underwent radiotherapy because of recurrent tumor growth in the follow-up (mean 26 months, range 12–71 months after the operation).

A postoperative hemorrhage occurred in three cases, which led to a revision in two cases. Postoperative hydrocephalus, including the patients with a postoperative hemorrhage, with the need for external ventricular drainage, was observed in six cases, two of them needed further therapy by continuous drainage (ventricular peritoneal shunt). An intraoperative air embolism due to the semi-sitting position occurred in three cases, without the need to interrupt the operation. A postoperative CSF leakage was observed in six cases; a revision was performed in all cases. No CSF infection occurred. The mortality in our series was 0%; the overall morbidity was 10%.

### Illustrative Case

The resection was then completed and a minimal residual tumor was left at the entrance to the inner auditory canal. Postoperatively, the patient showed no facial paresis.

## Discussion

Treatment in VS consists of three strategies. Firstly, a “wait and scan” strategy is recommended in small tumors with no or less symptoms. Hearing preservation, audiometric control and a stable neurological status as well as stable magnetic resonance imaging controls without tumor growth is required for this strategy (3, 6). Secondly, radiosurgery in VS for smaller and medium size tumors and hearing deterioration is a treating option (15, 16). Long-term follow-up showed stable hearing preservation for the first 2 years after treatment up to 80% which decreased to 23% after 10 years. Tumor control was up to 90% after 5 years (17–20). Thirdly, microsurgical resection in large tumors with dislocation of the cerebellum or brain stem is preferred after failed radiosurgery or in younger patients. The surgical approach, in the case of particularly large tumors, is to achieve mass reduction and reduce pressure even of the brainstem without restricting facial nerve function.

One of the goals in microsurgery for VS is to prevent nerve injury and loss of facial nerve function that is accompanied by a decrease in the quality of life (12, 13). Therefore, the use of intraoperative neurophysiological monitoring has been obligatory for decades and should be performed for every operation in the cerebellar pontine angle (8). The detection and visualization of the facial nerve or the fascicles of the nerve in larger tumors is sometimes difficult when the nerve is sprawled out in the tumor capsule. In addition, profuse hemorrhage and loss of the pial border can influence the visualization of the nerve routs. Direct nerve stimulation and EMG monitoring in the tumor capsule can subsequently provide nerve injury. In some cases, repeated stimulation with a drop in the EMG signal also leads to a smaller extent of resection, especially in the case of stronger adhesions. This of course results in the fact that in the case of incomplete resection, the preoperative size of the tumor as well as the size of the residual tumor have an influence on the function of the facial nerve (21–24). The aim of this study was to analyze influencing parameters according to postoperative facial nerve function and the use of intraoperative neurophysiological monitoring for the guidance of tumor resection. Various surgical approaches and patient positions for the microsurgical resection of VS are known and the retrosigmoidal craniotomy in a semi-sitting or park bench position is preferred in many centers (25). However, the complication rates for neurosurgical resection are low, as could be shown in the present study, 10 % morbidity, which is equal to the published literature (26–28). The operative strategy in our clinic is function-oriented with the aim of neurological integrity. If the aim of the operation is the most radical possible resection, then, as described in the literature, the size will very well have a significant influence. Conversely, we have shown that even large tumors can be surgically treated without a significant deterioration in facial function.

Functional orientated resection might be associated with a higher number of near total or subtotal resections. On the other hand, residual tumors contain the risk of regrowth and even the follow-up of residual tumor shows a stable tumor situation in over 65% of cases (10, 20, 29, 30). In our experience, the function of the facial nerve can be reliably classified after a year, after which the neurological condition usually does not change.

New strategies were implemented for a stepwise treatment to prevent nerve injury. First, subtotal or near total resection, if possible, with secondary radiosurgery (31). This strategy might be examined in future studies.

In our study, the postoperative facial nerve function and the amplitude of the direct stimulation of the facial nerve in the orbicularis oris muscle showed significance (*p* = 0.03); this was also shown in the multivariate analysis. This means that a direct nerve stimulation during the operation with a decreased amplitude while under stimulation could show a loss of nerve function. The repeated stimulation of the tumor surface to detect the facial nerve, which is often rolled out and difficult to visualize, especially in large tumors, leads to a lower proportion of injuries and thus paresis.

In the study, it was possible to analyze multivariately that a drop in the amplitude of the EMG of the oris muscle in particular is significantly associated with a poorer outcome of facial nerve function. Repeated intraoperative stimulation with the observation of a drop in amplitude can therefore indicate that the nerve has been damaged or that there is a risk.

In a recently published meta-analysis by Quimby et al. (32) the critical amplitude <500 μV of the EMG in direct nerve stimulation had sensitivity of 0.87 (95% CI.78– 0.93) for poor short-term facial function. This agrees with our data. The future will show the extent of further technical development, as shown by Matsushima et al. (33).

Amano et al. (8) also reported the correlation between the amplitude of the direct nerve stimulation and the postoperative facial nerve function. Therefore, the repeated stimulation of the facial nerve is advisable. The HBS was not influenced concerning the extent of resection. In our series, with the aim of neurological protection under EMG monitoring, no significant connection between facial nerve function and the extent of resection could be demonstrated. This is certainly also explained by the fact that the resection remained rather limited with a worsened facial nerve EMG. If the nerve could be prepared well and the EMG showed no seizures, a complete resection was also performed. Conversely, one can postulate that with function-oriented care, the Facilais function is not significantly endangered even in large tumors.

Patient parameters, special tumor items, and complications showed no influence in the postoperative facial nerve function in the follow-up either.

## Conclusion

Intraoperative neurophysiological monitoring and direct nerve stimulation for facial and trigeminal nerve electromyography is crucial in microsurgery for cerebellar pontine angle processes. In our series, deterioration of the amplitude in facial nerve stimulation was associated with postoperative facial nerve functional loss. Therefore, the repeated direct stimulation could indicate the risk of nerve damage.

## Data Availability Statement

The raw data supporting the conclusions of this article will be made available by the authors, without undue reservation.

## Ethics Statement

The studies involving human participants were reviewed and approved by Ethik-Kommission an der Medizinischen Fakultät der Universität Leipzig Liebigstraße 18 04103 Leipzig. Written informed consent for participation was not required for this study in accordance with the national legislation and the institutional requirements.

## Author Contributions

All authors listed have made a substantial, direct, and intellectual contribution to the work and approved it for publication.

## Conflict of Interest

The authors declare that the research was conducted in the absence of any commercial or financial relationships that could be construed as a potential conflict of interest.

## Publisher's Note

All claims expressed in this article are solely those of the authors and do not necessarily represent those of their affiliated organizations, or those of the publisher, the editors and the reviewers. Any product that may be evaluated in this article, or claim that may be made by its manufacturer, is not guaranteed or endorsed by the publisher.
